# Expression of *Hox*, *Cdx*, and *Six3*/*6* genes in the hoplonemertean *Pantinonemertes californiensis* offers insight into the evolution of maximally indirect development in the phylum Nemertea

**DOI:** 10.1186/s13227-015-0021-7

**Published:** 2015-08-04

**Authors:** Laurel S Hiebert, Svetlana A Maslakova

**Affiliations:** Oregon Institute of Marine Biology, University of Oregon, Charleston, OR USA

**Keywords:** Maximally indirect development, Pilidium, Nemertea, Larval evolution, *Hox*

## Abstract

**Background:**

Maximally indirect development via a pilidium larva is unique to the pilidiophoran clade of phylum Nemertea. All other nemerteans have more or less direct development. The origin of pilidial development with disjunct invaginated juvenile rudiments and catastrophic metamorphosis remains poorly understood. While basal members of the phylum, the Palaeonemertea, do not appear to have ever had a pilidium, certain similarity exists in the development of the Pilidiophora and the sister clade, the Hoplonemertea. It is unclear whether this similarity represents the homology and whether pilidial development evolved before or after pilidiophorans diverged from hoplonemerteans. To gain insight into these questions, we examined the expression of *Hox*, *Cdx*, and *Six3*/*6* genes in the development of the hoplonemertean *Pantinonemertes californiensis* and expression of *Six3*/*6* in the pilidium of *Micrura alaskensis*. To further characterize the function of larval structures showing expression of these genes, we examined the serotonergic nervous system and cell proliferation in *P. californiensis*.

**Results:**

We show that *Hox* and *Cdx* genes, which pattern the pilidial imaginal discs giving rise to the juvenile trunk, are expressed in paired posterior epidermal invaginations in *P. californiensis* larvae. We also show that *Six3*/*6* patterns both the pilidial cephalic discs, which give rise to the juvenile head, and a pair of anterior epidermal invaginations in hoplonemertean development. We show that anterior invaginations in larval *P. californiensis* are associated with a pair of serotonergic neurons, and thus may have a role in the development of the juvenile nervous system. This is similar to the role of cephalic discs in pilidiophoran development. Finally, we show that four zones of high cell proliferation correspond to the paired invaginations in *P. californiensis*, suggesting that these invaginations may play a similar role in the development of the hoplonemertean juvenile to the role of imaginal discs in the pilidium, which also exhibit high rates of cell proliferation.

**Conclusions:**

Expression of *Hox*, *Cdx*, and *Six3*/*6* genes supports the homology between the imaginal discs of the pilidium and the paired larval invaginations in hoplonemerteans. This suggests that invaginated juvenile rudiments (possible precursors to pilidial imaginal discs) may have been present in the most recent common ancestor of the Pilidiophora and Hoplonemertea.

**Electronic supplementary material:**

The online version of this article (doi:10.1186/s13227-015-0021-7) contains supplementary material, which is available to authorized users.

## Background

Development in nemerteans, a phylum of mostly marine predatory worms, ranges from direct with a crawl-away juvenile, to mostly direct with a planktonic juvenile-like larva, to a maximally indirect with a unique planktonic larva known as the pilidium [1, 2]. The pilidium is a feeding larva, which has a distinctly different body plan from that of the adult. The juvenile develops inside the pilidium from a set of initially isolated rudiments called imaginal discs [see 3 and references therein]. The imaginal discs grow and fuse together around the larval stomach over a span of weeks or months. Once the juvenile is completely formed inside the pilidium, the larva undergoes a catastrophic metamorphosis, in which the juvenile escapes from and devours the larval body [3–5]. The pilidial life cycle is found in a single clade of nemerteans, the Pilidiophora [6, 7]. However, when, in relation to the major nemertean lineages, and how this metamorphic life history arose remains unclear [2].

The basal lineage, Palaeonemertea, exhibits no traces of pilidial development. Palaeonemerteans typically possess so-called planuliform (superficially planula-like) planktonic larvae that develop into juveniles without a conspicuous metamorphosis. Maslakova et al. [8, 9] found that the larva of the paleonemertean *Carinoma tremaphoros* has a “hidden” prototroch derived from the classical spiralian trochoblast lineage, and this prototroch is lost early in larval development. This suggests that a kind of trochophore larva (such as found in related spiralian phyla, e.g., annelids and mollusks) may have characterized ancestral nemerteans. The pilidium, on the other hand, appears to have evolved within nemerteans, after the split between the Palaeonemertea and the Neonemertea, which comprise the Hoplonemertea and the Pilidiophora [6, 7, 10].

Similar to the palaeonemerteans, the Hoplonemertea, the sister clade to the Pilidiophora, develop via a planuliform larval stage, which may be encapsulated or free-swimming [1, 2, 11]. However, the larvae of hoplonemerteans differ from those of palaeonemerteans in a number of ways. None of the studied hoplonemertean larvae have a recognizable prototroch (such as was found in *Carinoma*). Instead, they have features that are rather reminiscent of pilidia. Many species were found to have a transitory larval epidermis composed of large ciliated cells that are gradually displaced (resorbed or shed) by the much smaller cells of the definitive epidermis [12, 13]. This loss of larval epidermis may be homologous to pilidial metamorphosis [2, 14, 15]. Maslakova [2] coined the term “decidula” for the hoplonemertean larvae to emphasize the process of ontogenetic loss of the larval epidermis. Some decidulae have been noted to have bilaterally symmetrical epidermal invaginations whose fate or function is not always clear (Maslakova and von Döhren [12]). Hiebert et al. [13] hypothesized that some of these invaginations may be homologous to imaginal discs of the pilidium. Thus, it is possible that pilidial development, in some form, has evolved before the split between the Hoplonemertea and the Pilidiophora, and these invaginations represent vestiges of imaginal discs. Alternatively, pilidial development may have evolved at the base of the Pilidiophora, from the kind of development found in extant hoplonemerteans, and these structures in hoplonemertean development represent precursors to pilidial imaginal discs. Regardless of which is correct, until now we lacked other evidence of homology beyond vague morphological similarity.

Here, we provide additional evidence of homology between pilidial imaginal discs and paired invaginations of the hoplonemertean decidula based on the expression of the axial patterning genes *Hox* and *Cdx* in the hoplonemertean species *P. californiensis* and expression of *Six3*/*6* in pilidial and hoplonemertean development. To better illustrate organogenesis, larval anatomy, and to understand the function of these invaginations in hoplonemertean development we also examined the structure of the serotonergic nervous system and cell proliferation patterns in *P. californiensis* larvae.

## Methods

### Collection of adults and larval culturing

Adult hoplonemerteans, *P. californiensis*, were collected under rocks along the high intertidal above a mudflat in Coos Bay near Glasgow, OR. Reproductive adults were identified in the field, as gametes are visible through the body wall (pink oocytes, white sperm), and transported in 50-mL plastic tubes to the Oregon Institute of Marine Biology in Charleston, OR. Adults were kept inside individual 50-mL conical tubes with a few milliliters of 0.45 µm-filtered sea water (FSW) in the flowing sea-water table at ambient sea temperature. Water was changed every few days. Spawning, in some cases, was observed immediately after collection, or days to weeks later following a water change. But spawning also occurred without any observed change in conditions. Gametes were successfully obtained July–September of 2009–2013. Our observations of spawning suggest that timing may be influenced by phase of the moon, but we have not rigorously tested this hypothesis. On a number of occasions, the worms released gametes during 2–3 days around the time when the absolute heights of the two daily low tides were similar to each other. Spawned oocytes were resuspended in FSW in glass custard dishes and fertilized with a few drops of dilute sperm suspension. Fertilized eggs were kept at ambient seawater temperature (12–16°C). After hatching (1 day post-fertilization) swimming larvae were transferred into a clean custard dish with fresh FSW. Water was changed every few days by reverse filtration. Collection, fertilization, and larval culture methods for the pilidiophoran *Micrura alaskensis* were as described in Maslakova [3] and Hiebert and Maslakova [16].

### Isolation and identification of *Hox*, *Cdx*, and *Six3*/*6* sequences

Sequences of developmental patterning genes were identified in developmental transcriptomes of *P. californiensis* and *M. alaskensis* prepared by us (Meyer et al. in prep., Hiebert and Maslakova, unpublished). The *P. californiensis* transcriptome was prepared using cDNA from seven developmental stages (invagination stage/24 h post-fertilization, late-invagination stage/48 h, early-rudiment stage/3 days, late-rudiment stage/4 days, late-rudiment stage/5 days, early-vermicular stage/7 days, and vermicular stage/2 weeks). The transcriptome for *M. alaskensis* also contained transcripts from seven developmental stages (gastrula, young feeding pilidium, cephalic-disc stage, cerebral-organ-disc stage, head-and-trunk stage, and hood-to-pre-metamorphosis stage). We refer to developmental stages alone and not the absolute age of pilidia, because the developmental rate is highly dependent on culturing conditions (temperature, feeding regime, food quality, and, possibly other factors) and development after the first week can be quite asynchronous even in a single culturing vessel. Sequences were isolated from cDNA using a combination of PCR and rapid amplification of cDNA ends [RACE, see 17] with primers designed using transcriptome contig sequences (Table 1). PCR and RACE products were sub-cloned into PGEM-t (Promega) vectors and then transformed into One Shot Top10 chemically competent *E. Coli* cells (Invitrogen). DNA from clones was purified (QIAprep Spin Miniprep Kit, Qiagen) and sequenced in both forward and reverse directions on an ABI 3730xl DNA Analyzer platform (Sequetech, Mountain View, CA, USA) using T7 and SP6 primers.

**Table 1:** Primers used for *Hox* amplification

Gene name	Primer 1	Primer 2
PcLab	Nested RACE (5′ end): CGACGTGGACTATCCATGAACGCAAAGCAGTGGTATCAACGCAGAGT TCGAGCGGCCGCCCGGGCAGGTCGACGTGGACTATCCATGAACGCA	Nested RACE (gene specific): ACCTCCAGGTTGGGCATTGACCTT TCGTTTAGTCCACGGCCACCTCAT
PcPb	CACACGGAGATTCCGGAGAGAGGG	GTGGGGACCATTCACATTTCTACCGA
PcHox3	ACCCGACCACGTGACTGACTTCAA	AGCAAGCGTGCACCATACAGTTCC
PcDfd	AGCCGATCCTCAATGGTCGGTGAA	ATTCACTCGCAGCTCGCAGGAAAA
PcLox5	ACTTGGCATGACTTGCTGACATGGT	TGGAAACGAGCAAACGGTGGTAGC
PcPost2	TCTGCTACGGGCATCAGTTGCCTA	CAGGGCAGAGTGACCAGACCTACC
PcCdx	TCTGCTACGGGCATCAGTTGCCTA	CAGGGCAGAGTGACCAGACCTACC

### Orthology assessment

*Hox* and *Cdx* gene orthology was determined by phylogenetic analysis. *Hox* and *Cdx* fragments from *P. californiensis* were aligned with *Cdx* and *Hox* complements of a deuterostome (*Branchiostoma floridae)*, two ecdysozoans (*Tribolium castaneum*, *Drosophila melanogaster*), and five lophotrochozoans (an annelid *Capitella teleta*, a bryozoan *Bugula turrita*, a nemertean *Micrura alaskensis*, a brachiopod *Lingula anatina*, and a mollusk *Euprymna scolopes*) (see Additional file 1: Table S1). Bayesian inference analysis was conducted using MrBayes version 3.2.1 [18, 19]. *Hox* fragments were aligned using the homeodomain and the 12 3ʹ amino-acids since this upstream flanking region shows some sequence conservation. The analysis was done with the Rtrev amino-acid model with a gamma-shaped distribution of rates across sites. *Drosophila melanogaster Even skipped* (*eve*) was specified as the outgroup. The analysis was done with five heated chains with 5,000,000 generations and was sampled every 500 generations. Four independent runs were conducted. The first 25% samples from the cold chain were discarded as burn-in. Trees were visualized and manipulated using FigTree version 1.4.0 and Adobe Illustrator version 17.1.0.

Phylogenetic analysis was also performed to determine *Six3*/*6* gene orthology. *Six* family sequences from representative taxa were retrieved from NCBI (http://www.ncbi.nlm.nih.gov/; accession numbers listed in Additional file 1: Table S2), including those from three deuterostomes (*Mus musculus*, *Saccoglossus kowalevskii*, *Strongylocentrotus purpuratu*s), one ecdysozoan (*Drosophila melanogaster*), three lophotrochozoans (a brachiopod *Terebratalia transversa*, a mollusk *Lottia gigantea*, the annelids *Capitella teleta* and *Platynereis dumerilii*), and a cnidarian (*Nematostella vectensis*). These sequences were aligned with the *Six3*/*6* sequences from *M. alaskensis* and *P. californiensis*, using MUSCLE v3.8 [20]. Alignment was checked by eye. Bayesian analysis and tree visualization was conducted as described above.

### Larval fixation and in situ hybridization

Prior to fixation, larvae were relaxed in 1:1 mix of 0.37 M MgCl_2_:FSW for 10 min. Larvae were fixed overnight at 4°C in 4% paraformaldehyde made up from 16% paraformaldehyde (Electron Microscopy Sciences) in FSW, then washed three times in 1× phosphate-buffered saline, pH 7.4 (PBS, Fisher Scientific) with 0.2% Triton X-100 (Fisher Scientific). Larvae were then washed twice with deionized water, dehydrated in a series of methanol (25, 50, 75, 100%), and stored at −20°C in 100% methanol. Probe preparation and in situ hybridization was conducted as described in Hiebert and Maslakova [16]. In short, larvae were rehydrated in PBS, acetylated with triethanolamine and acetic anhydride, re-fixed with paraformaldehyde, and hybridized in a formamide buffer with 1 ng/µl dioxygenin (DIG)-labeled RNA probe at 63°C for 2–3 days. Excess probe was washed away in low concentration saline sodium citrate. The remaining bound probe was labeled with anti-DIG alkaline phosphatase, which was allowed to react in the dark with Nitro Blue Tetrazolium and 5-bromo-4-chloro-3-indolyl phosphate for 1 h to overnight until blue-purple color developed. Larvae were mounted in 80% glycerol in PBS. Larvae were imaged with a Leica DFC400 digital color camera mounted on an Olympus BX51 microscope equipped with differential interference contrast optics. Ten to twenty specimens were examined for each gene and stage. Some in situ-stained larvae were mounted with propidium iodide in 80% gylcerol in 1× PBS on poly-l-lysine-coated coverslips for confocal microscopy. Coverslips were dipped into a 0.1% poly-l-lysine (Sigma) solution and allowed to dry.

### Antibody staining

Larvae were fixed as above and rehydrated from methanol into phosphate-buffered saline (PBS) via 5-min changes in 60% methanol, 30% methanol, and then PBS. Larvae were permeabilized with three 10-min washes in PBS with 0.1% Triton X-100 (PBT). To block non-specific staining, larvae were incubated in 5% normal goat serum in PBT for 2 h at room temperature. Normal goat serum was washed out with three 10-min washes in PBT. Larvae were incubated overnight at 4°C in one of two primary antibodies diluted 1:500 in PBT: rabbit-anti-5HT (Immunostar, Cat #20080) or rabbit-anti-phospho-histone H3 (Ser10) (Millipore, Cat #06-570). Larvae were then washed in three 10-min changes in PBT and incubated for 2 h at room temperature in goat-anti-rabbit 488 secondary antibody (Molecular Probes, Cat #A11008) diluted 1:600 in PBT. Larvae were then washed in three 10-min changes of PBS and mounted on poly-l-lysine-coated coverslips using CFM-2 mounting media (CitiFlour LTD).

### Confocal microscopy and analysis

Confocal images were obtained with an Olympus FluoView 1000 laser scanning confocal system (Olympus America, Center Valley, PA, USA) mounted on an Olympus IX81 inverted microscope with a UPlanFLN 40 × 1.3 NA oil lens or a PlanApoN 60× 1.42 NA oil lens. Images were processed with ImageJ v. 1.45b (Wayne Rasband, National Institutes of Health, Bethesda, MD, USA). Overlays were made using Photoshop CC (2014, Adobe).

## Results

### Development of *P. californiensis*

**Fig. 1:**
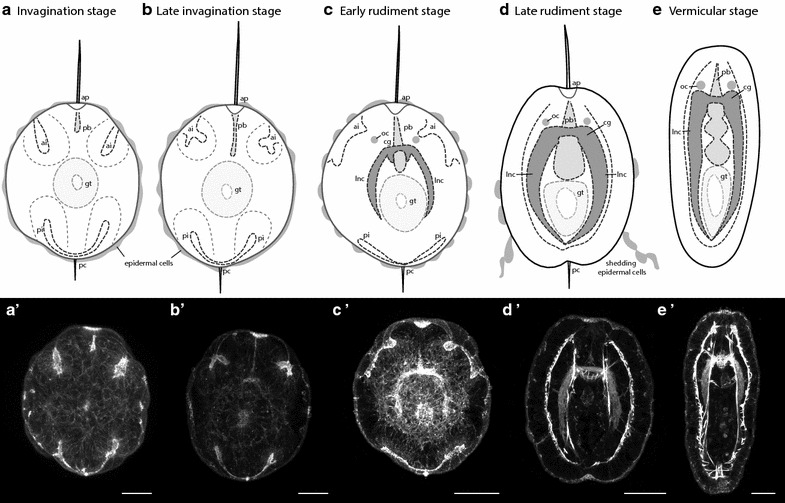
Diagram of developmental stages of *P. californiensis.* **a** Invagination stage, covered with large epidermal cells. **b** Late-invagination stage, in which anterior invaginations have bifurcated. **c** Early-rudiment stage. **d** Late-rudiment stage, in which epidermal cells are shed in chains. **e** Vermicular stage. *ap* apical organ, *pb* proboscis, *gt* gut, *pc* posterior cirrus, *ai* anterior invagination, *pi* posterior invagination, *cg* cerebral ganglia, *oc* ocellus, *lnc* lateral nerve cord. **a′**–**eʹ** Confocal projections of phalloidin-labeled larvae modified from Hiebert et al. [13]. **a′** A 30-μm-thick sub-stack of frontal sections of invagination-stage larva. **b′** A 29-μm-thick sub-stack of frontal sections of late-invagination-stage larva. **c′** A 15-μm frontal sub-stack of an early-rudiment-stage larva. **d′** A 5-μm sub-stack of frontal sections of a late-rudiment-stage larva. **e′** An 8-μm sub-stack of frontal sections of a vermicular-stage larva. *Scale bars* 25 µm.

Larval development of *P. californiensis* is described in detail in Hiebert et al. [13] and will be briefly summarized here. Adults spawn either sperm or oocytes. Once fertilized, the eggs undergo spiral cleavage and begin to develop cilia by 22 h at 13–14°C. Uniformly ciliated bullet-shaped larvae equipped with an apical tuft hatch out of the chorion at around 30 h. Larvae at this stage (called “invagination stage”) possess a mouth, an unpaired proboscis rudiment invagination, and two paired invaginations: an anterior and a posterior pair (Fig. 1a). The epidermis of the invagination-stage larva comprises ~80 large multiciliated cells (Fig. 1a). These cells constitute the transitory larval epidermis. By 2 days, the paired anterior invaginations appear to bifurcate (Fig. 1b) and by 3 days to evaginate. At this time, smaller cells of the definitive epidermis begin to emerge between the larger cells of the embryonic epidermis separating the large cells (Fig. 1c). By this time, larvae also have two brown ocelli, a distinct proboscis rudiment, and rudiments of the cerebral ganglia, cerebral commissures, and lateral nerve cords. This stage is referred to as the “early-rudiment” stage. By “late-rudiment” stage, larvae shed large multiciliated cells of the transitory larval epidermis, often several at a time, leaving them behind connected in a chain (Fig. 1d). The larval epidermis is replaced by the smaller cells of definitive epidermis. By “vermicular” stage, the larva has elongated and exhibits worm-like behaviors (Fig. 1e).

### Serotonergic nervous system in *P. californiensis* larvae

The central nervous system of rudiment-stage *P. californiensis* larvae is a blueprint of the adult nervous system. It consists of paired cerebral ganglia (two dorsal and two ventral) joined into a brain ring around the proboscis via ventral and dorsal cerebral commissures, and the two lateral nerve cords. Lateral nerve cords originate from the ventral cerebral ganglia. The nervous system occupies the majority of space between the body wall (epidermis + body wall muscles) and the gut. Phalloidin labeling helps to visualize the fibrous core (consisting of axons) of the cerebral ganglia and the lateral nerve cords, but not the ganglionic portion (i.e., the cell bodies), which surrounds the fibrous core (Fig. 1c–e). To help visualize at least some of the cell bodies, here we describe the serotonergic component of the nervous system.

**Fig. 2:**
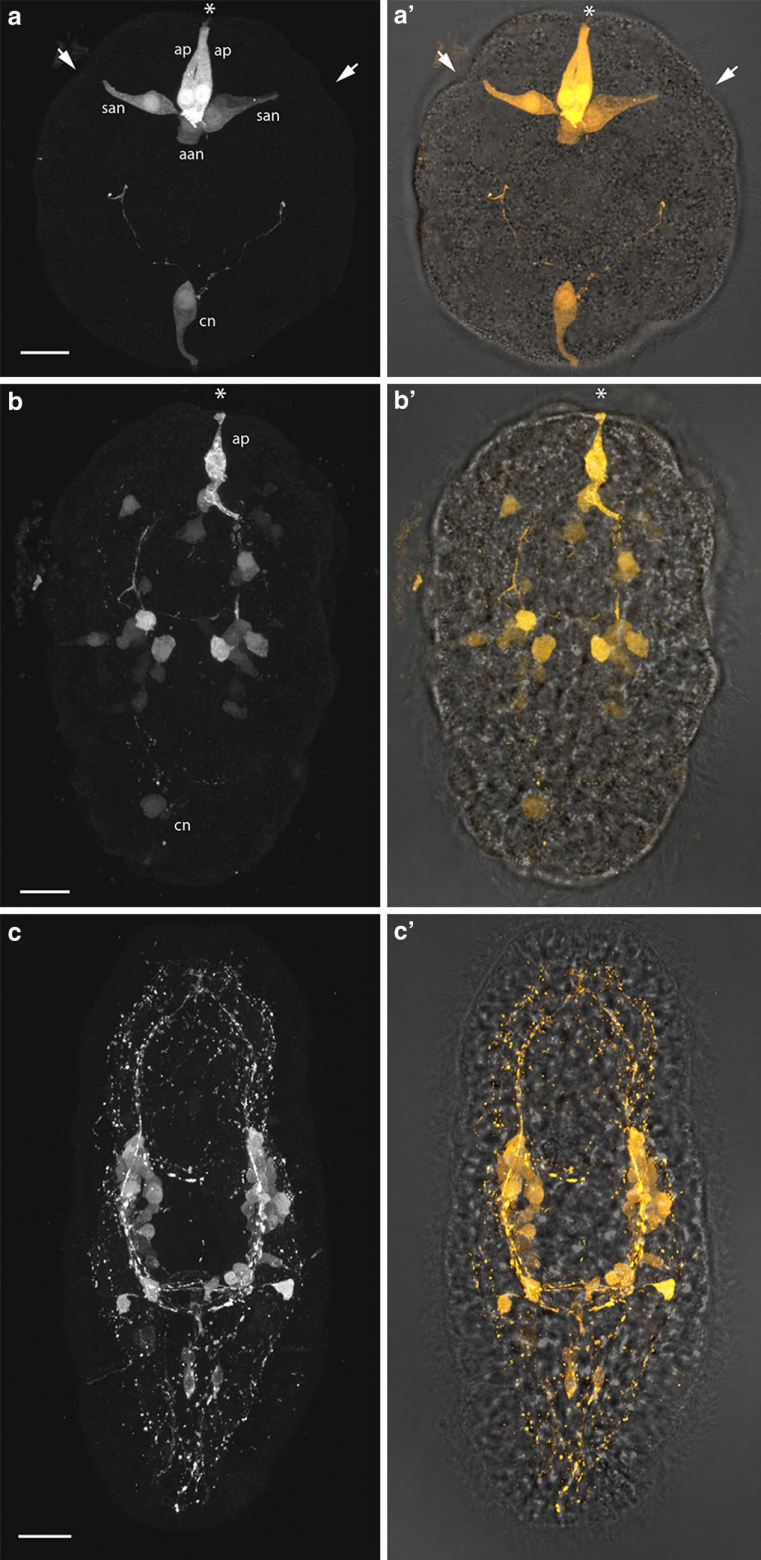
Serotonergic nervous system of *P. californiensis* larvae. Larval anterior is up on all panels. **a** Confocal *z*-projection of anti-serotonin antibody labeling in invagination-stage larva. Larva possesses seven serotonergic neurons, two of which appear to innervate the anterior invaginations. Neuron labels: *ap* apical neuron, *san* subapical neuron, *aan* additional apical neuron, *cn* caudal neuron. *Asterisk* indicates apical organ. **aʹ** Overlayed fluorescent and transmitted-light image. **b** Confocal *z*-projection of anti-serotonin antibody labeling of the early-rudiment-stage larva. **b′** Overlayed fluorescent and transmitted-light image. **c** Confocal *z*-projection of anti-serotonin antibody labeling of late-rudiment-stage larva. **c′** Overlaid fluorescent and transmitted-light image. *Scale bars* 15 µm.

Invagination-stage *P. californiensis* larvae have seven serotonergic neurons (Fig. 2a). Anteriorly, there is a closely apposed pair that appears to be a part of the apical organ of the larva. These are referred to as the apical neurons [21]. A second pair of so-called subapical neurons is found ventrolateral to the apical neurons (Fig. 2a). These subapical neurons have processes that end at epidermis close to the anterior invaginations. Two so-called additional apical neurons are connected to both the apical and subapical neurons and are found just posterior to the apical neurons. Lastly, an unpaired neuron is situated at the posterior end of the larva and has dendritic connections to the caudal epidermis. This caudal neuron also has two long bifurcating processes extending toward the anterior end of the larva (Fig. 2a).

Early-rudiment-stage larvae have apical neurons, a caudal neuron, and a number of additional neurons in the regions of the developing cerebral ganglia and lateral nerve cords (Fig. 2b). By late-rudiment stage, larvae have lost the apical and caudal neurons (Fig. 2c). Most serotonergic neurons are situated laterally along the developing lateral nerve cords at this stage (Fig. 2c). The serotonergic neurons form a connection between the lateral nerve cords in the posterior half of the larva. Additional paired neurons are present posterior to and lateral to this connection (Fig. 2c).

Although serotonergic neurons comprise only one component of the nervous system, they illustrate the position and relative proportion of the nervous system elements in the larval body, specifically the two large groups of cells at the anterior portion of the lateral nerve cords. This is important because we later describe the expression of several gene markers in these regions.

### *Hox*, *Cdx*, and *Six3*/*6* sequences

Six *Hox*-containing contigs and one with *Cdx* were recovered from the developmental transcriptome of *P. californiensis*. Long coding sequences were isolated for all seven genes. Based on Bayesian phylogenetic analysis (see Additional file 2), *P*. *californiensis* has *Hox* gene representatives from six paralog groups (PGs): PG1 (*Labial*/*Lab*), PG2 (*Proboscipedia*/*Pb*), PG3 (*Hox3*), PG4 (*Deformed*/*Dfd*), PG6 (*Lox5*), and PG9–PG13 (*Post2*). The paralog group, length of predicted open reading frame, and GenBank accession number for each gene are listed in Additional file 1: Table S3. Both the *M. alaskensis* and *P. californiensisSix* family genes recovered from the respective transcriptomes fall into the *Six3*/*6* clade (see Additional file 3).

### *Hox* and *Cdx* expression in the development of *P. californiensis*

**Fig. 3:**
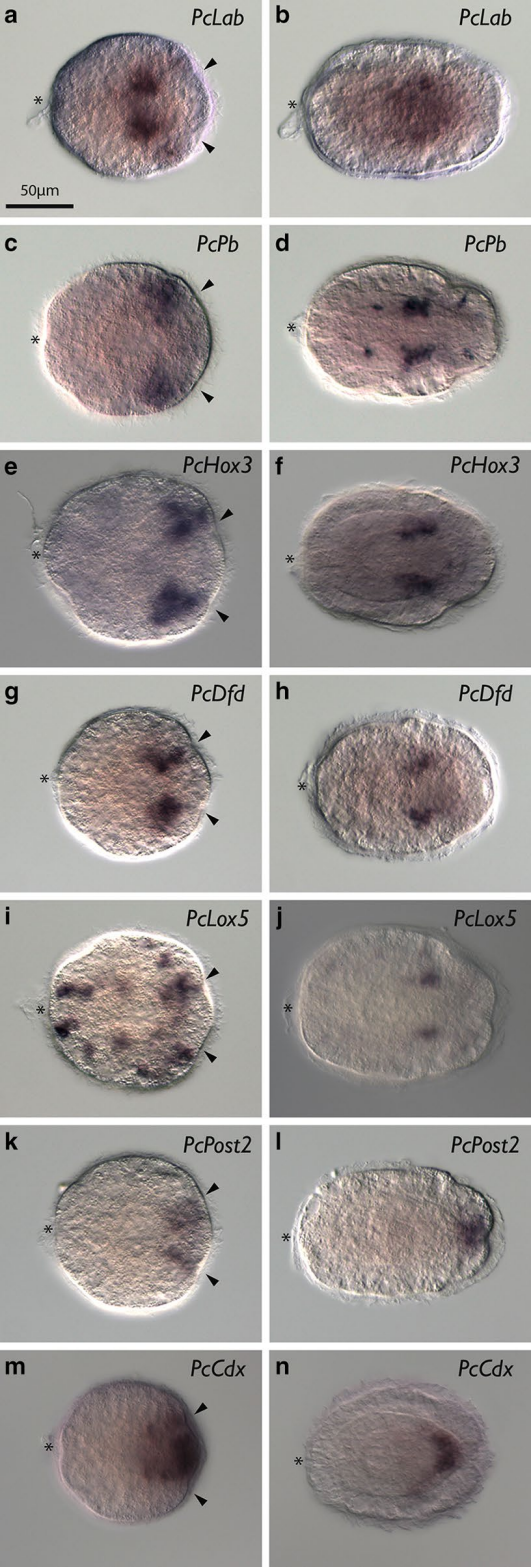
*Hox* and *Cdx* expression in *P. californiensis*. All images show frontal views with larval anterior to the *left.* *Left column* shows invagination-stage larvae. *Right column* shows rudiment-stage larvae. *Arrowheads* indicate paired posterior invaginations. *Asterisk* indicates apical organ.

**Fig. 4:**
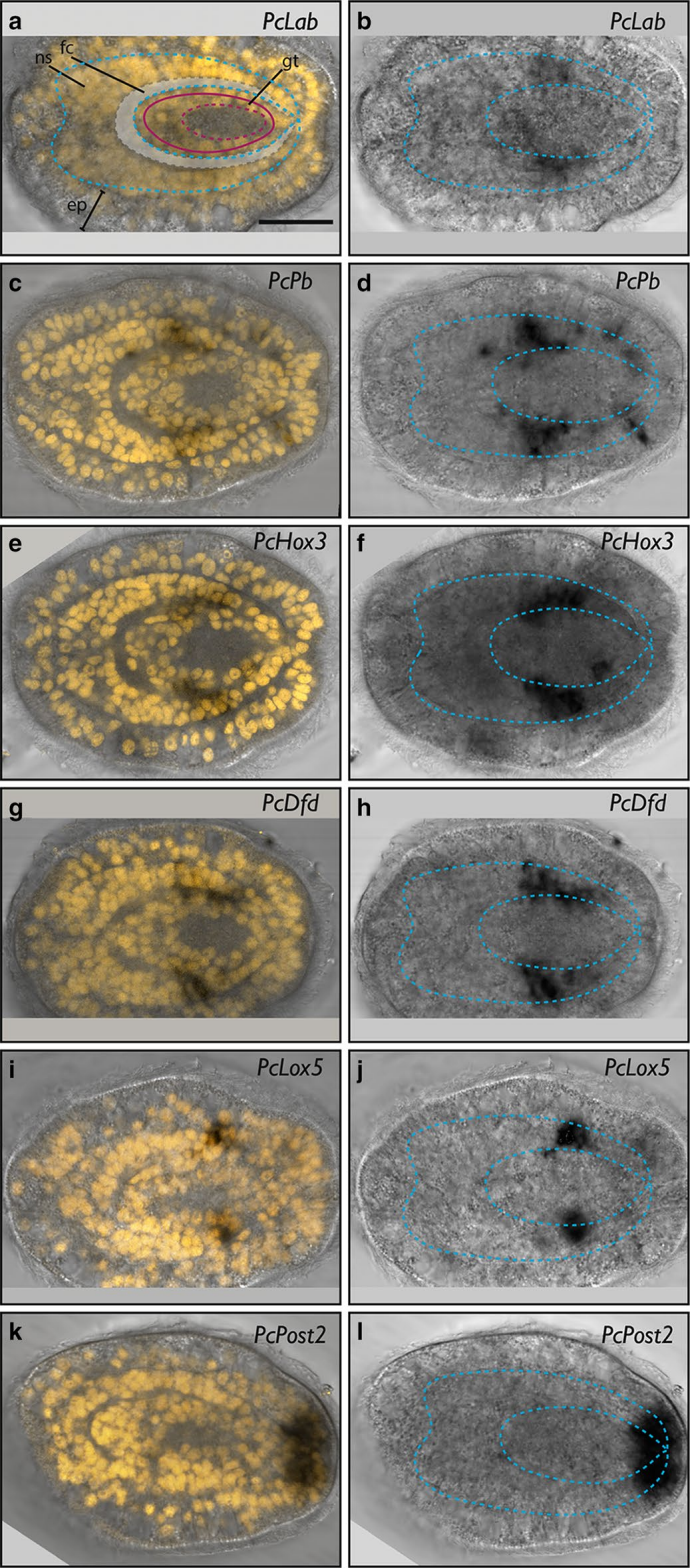
Confocal projections showing *Hox* gene expression in rudiment-stage larvae of *P. californiensis*. *Left panels* show nuclear staining (propidium iodide, in *yellow*) as an overlay over transmitted light images. Larval anterior is to the *left*. Outline of the central nervous system indicated by *blue dotted line* (ns). *Panel* **a** shows additional features, such as epidermis (ep), gut (gt) with lumen in *dotted line* (both in *red*), and fibrous core of cerebral ganglia and lateral nerve cords (fb, *gray shading*). *Right column* shows transmitted light images showing the relative position of the nervous system with *blue dotted line*. *Scale bar* is 20 µm.

**Fig. 5:**
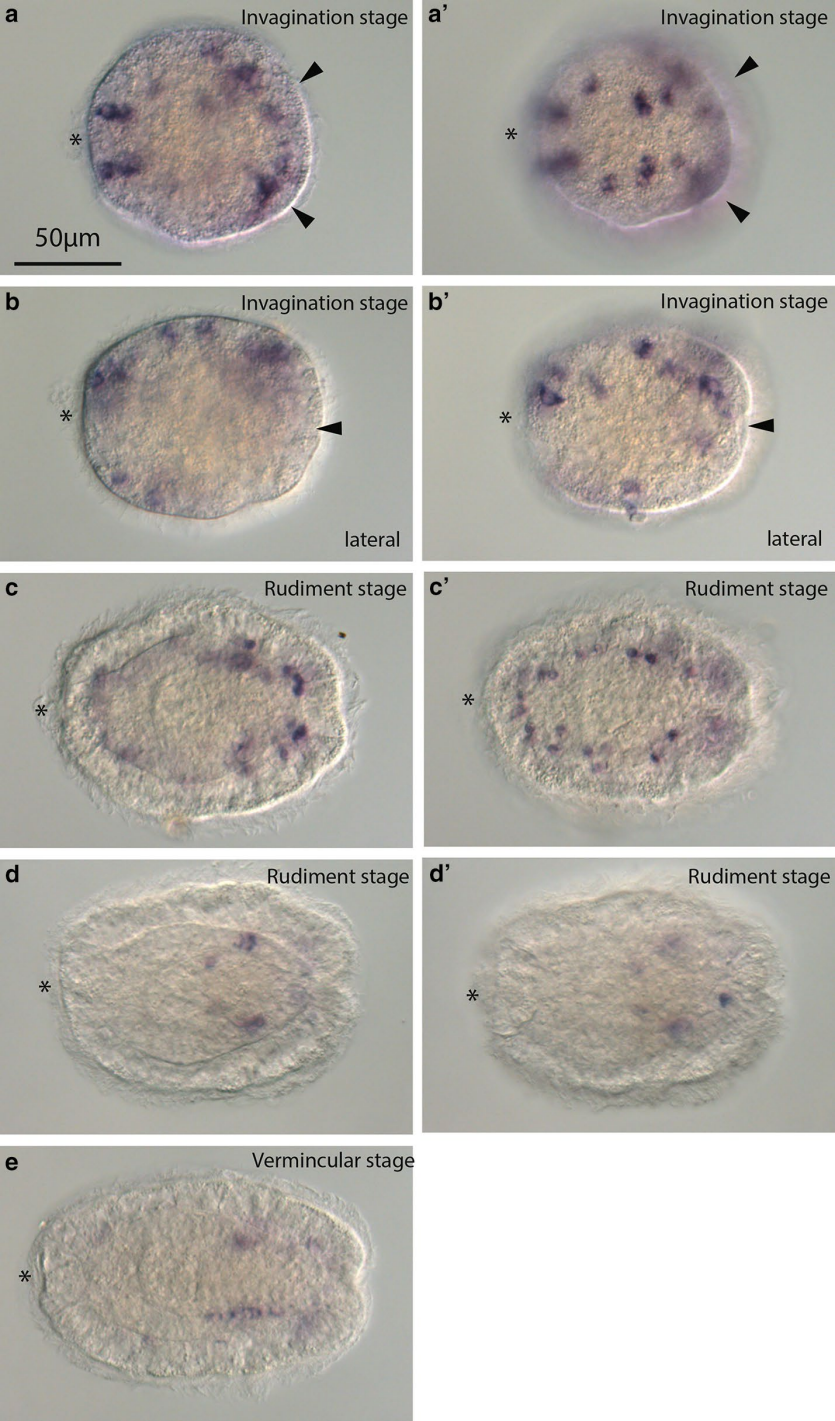
*Lox5* expression in the development of *P. californiensis.* Larval anterior is to the left. Stage of development is marked in the *upper right corner* of each image. Images in the *right column* show a different focal plane of the same larva as in the *left column*. Frontal views in all except **b** and **bʹ**. **b** and **bʹ** are lateral views. **a**, **b** Invagination-stage larva. **c** Rudiment-stage larva. **d** Late-rudiment stage. **e** Early-vermicular stage. *Arrowheads* indicate paired posterior invaginations. *Asterisk* indicates apical organ.

The expression of all six *Hox* genes and *Cdx* was examined at the invagination stage, early- and late-rudiment stage, and vermicular stage. At invagination stage, *PcLab* showed expression in two bilaterally symmetric subepidermal patches close to the lateral midline (Fig. 3a). For this gene and others, it is difficult to determine exactly in which tissue expression occurs at the invagination stage. Most appear to be expressed subepidermally, but it is not always possible to differentiate epidermal expression in an invaginated region from subepidermal expression. By early-rudiment stage, the expression of *PcLab* is still subepidermal, but covers a larger domain in the posterior half of the larva (Fig. 3b). Double staining with the nuclear dye propidium iodide indicates that *PcLab* staining occurs in the cells of the central nervous system, specifically the lateral nerve cords (Fig. 4a, b). The fibrous cores of the nerve cords (composed of axons) are evident as regions without nuclear staining. *PcPb* is expressed more posteriorly and laterally than *PcLab* at the invagination stage (Fig. 3c). By rudiment stage, *PcPb* shows two relatively large bilaterally symmetrical subepidermal domains in what appears to be the lateral nerve cords (Fig. 4c, d) and four additional smaller patches—two on each side, one pair just anterior to and one just posterior to the broader domain (Fig. 3d). Based on their position, the anterior patches are likely neurons in the cerebral ganglia (Fig. 4d). Posterior patches, on the other hand, are clearly in the epidermis (and could also be neurons). Although we do not have double in situ hybridization data, *PcHox3* expression appears to overlap with *PcPb* at the invagination stage at least somewhat, but appears to be slightly more posterior (Fig. 3e). Similarly, at the rudiment stage the two *PcHox3* domains appear to overlap with the two large domains of expression of *PcPb* in the lateral nerve cords (Figs. 3f, 4e, f). At the invagination stage, *PcDfd* shows two subepidermal domains of expression similar to those of *PcPb* and *PcHox3* (Fig. 3g). At the rudiment stage, expression of *PcDfd* is quite similar to that of *PcHox3*, but with a slightly more restricted domain along the AP axis (Figs. 3h, 4g, h). *PcLox5* shows a pattern of expression different from the other genes at the invagination stage. Numerous small patches of expression are found both in the epidermis and subepidermally. Notably, two somewhat larger domains are found near the posterior; additionally, what appears to be individual cells show *PcLox5* expression in the anterior and lateral epidermis of the larva, as well as subepidermally (Figs. 3i, 5a, b). There appears to be dorsoventral asymmetry in the number of *PcLox5*-positive cells at this stage, but we cannot be sure whether it is the dorsal or the ventral side that shows more expression (Fig. 5b). *PcLox5* appears to be expressed in individual cells in the larval nervous system at early-rudiment stage (Fig. 5c), but is mostly restricted to two lateral patches of expression in the lateral nerve cords by the late-rudiment stage (Figs. 3j, 4i, j, 5d). At early-vermicular stage, *PcLox5* is expressed only weakly along what appears to be the lateral nerve cords (Fig. 5e). *PcPost2* expression occurs in two patches close to the posterior end of the invagination-stage larva (Fig. 3k). By rudiment stage, *PcPost2* shows a single patch of epidermal expression near the posterior end of the larva (Fig. 3l). *PcCdx* shows epidermal expression in a single domain at the posterior-most end of the invagination-stage larva (Fig. 3m) and in a smaller single subepidermal domain at the posterior end of the rudiment-stage larva (Fig. 3n).

### *Six3*/*6* expression in nemertean larvae

**Fig. 6:**
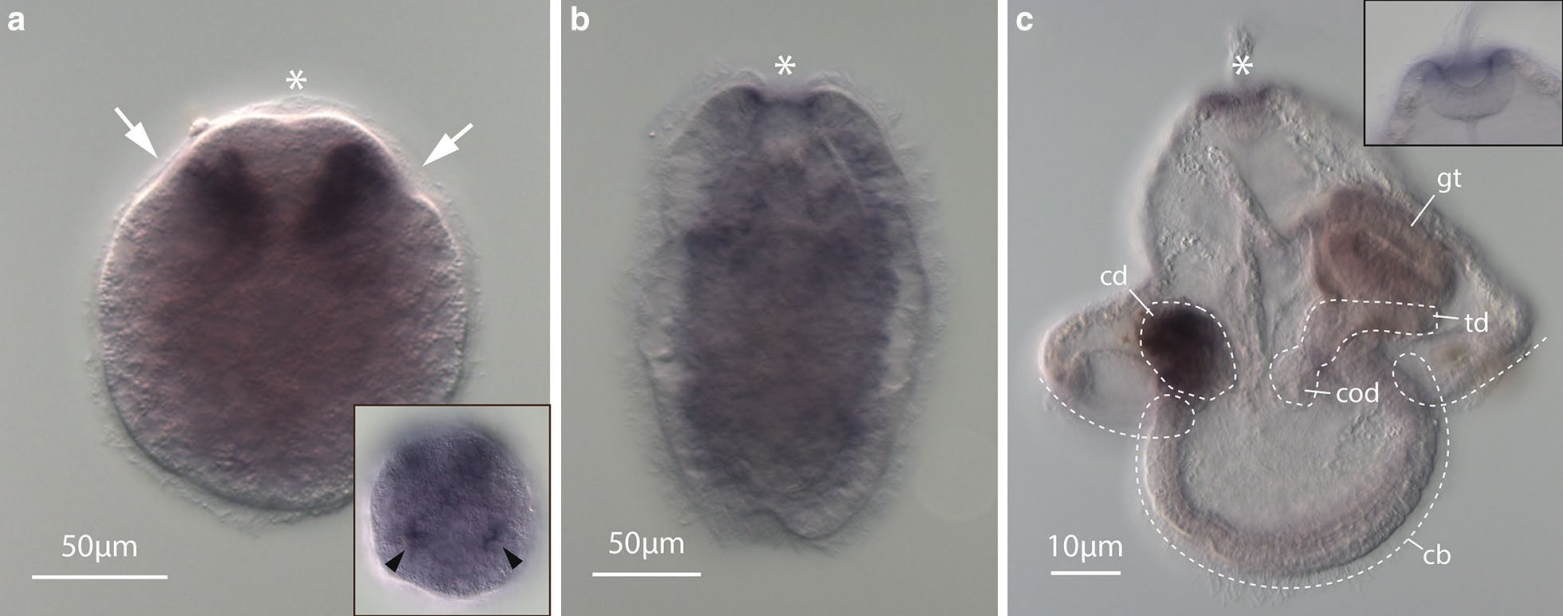
*Six3*/*6* expression in hoplonemertean and pilidiophoran larvae. **a** *Six3*/*6* is expressed in anterior invaginations of invagination-stage *P. californiensis* larva. *Arrows* indicate paired anterior invaginations. *Asterisk* indicates apical organ. *Inset* at *lower right* shows focal plane near the surface of another invagination-stage larva. Paired surface expression near posterior end marked by *black arrowheads*. **b** *Six3*/*6* expression in rudiment-stage larva, showing some diffuse subepidermal expression and strong expression near the apical plate. **c** *Six3*/*6* is expressed in cephalic discs (cd) and apical organ of head-and-trunk-stage *M. alaskensis* pilidium larva. *gt* gut, *cd* cephalic disc, *cb* ciliated band, *td* trunk disc. *Inset* shows close-up of mid-plane of the apical organ, showing expression along the rim of the apical plate.

In the hoplonemertean, *P. californiensisPcSix3*/*6* expression was examined in the invagination-stage and rudiment-stage larvae. At the invagination stage, the expression was evident in two distinct patches near the anterior invaginations (Fig. 6a), which are associated with serotonergic neurons (Fig. 2a). No strong expression was observed in rudiment-stage larvae (data not shown). In comparison, in the pilidiophoran *Micrura alaskensis*, the *Six3*/*6* ortholog is expressed in the cephalic imaginal discs of the pilidium larva (Fig. 6b). Additionally, there is expression near the pilidial apical organ (Fig. 6b). Hiebert and Maslakova [16] described expression of *MaSix3*/*6* in the additional developmental stages of *M. alaskensis*. Expression is first evident at the blastosquare stage in isolated cells. In young pilidia, *MaSix3*/*6* is expressed in several cells in the vicinity of the apical organ as well as the pilidial primary ciliary band [16, Supplementary Figure three]. These cells may correspond to the serotonergic neurons in pilidium larva [3]. Expression of *MaSix3*/*6* continues in the cephalic discs through the trunk-disc stage and weakens to a diffuse staining in the anterior end of the juvenile in later development (data not shown).

### Cell proliferation in the invagination stage *P. californiensis*

**Fig. 7:**
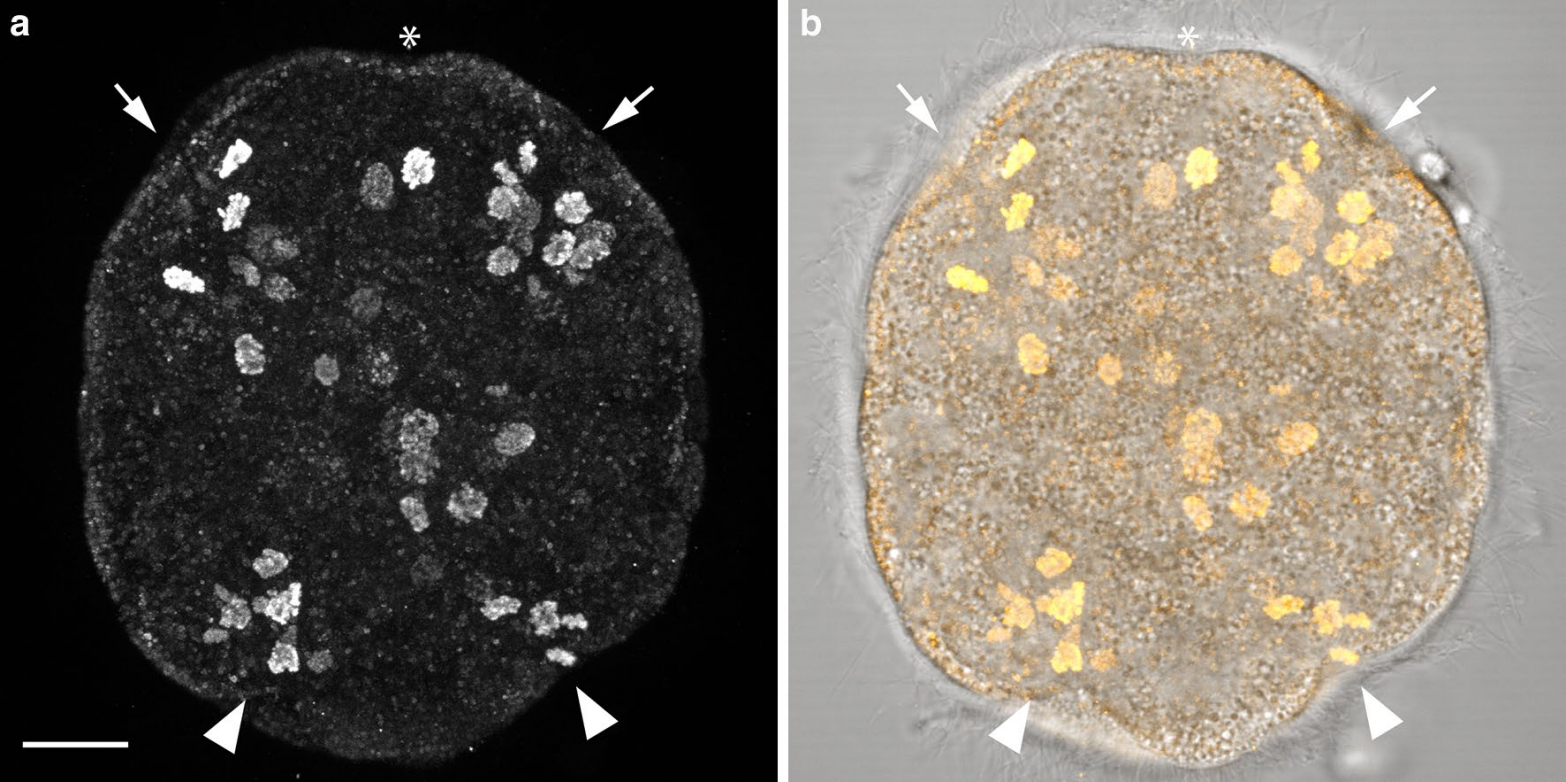
Cell proliferation patterns of *P. californiensis* invagination-stage larva. **a** Confocal *z*-projection of anti-phospho-histone antibody staining in invagination-state *P. californiensis* larva. **b** Overlaid fluorescent and transmitted-light image of the same larva. *Arrows* indicate the location of anterior invaginations. *Arrowheads* indicate the locations of posterior invaginations. *Asterisk* indicates apical organ.

Anti-phospho-histone antibody labeling was examined in the invagination-stage and rudiment-stage larvae of *P. californiensis.* Invagination-stage larvae possess four zones of dense labeling that appear to roughly correspond to the two pairs of epidermal invaginations (Fig. 7). Additional dividing cells are found in other regions of the larva, including a few near the apical organ and a number associated with the gut rudiment.

## Discussion

We isolated six *Hox* genes from *P. californiensis*: *Labial*, *Proboscipedia*, *Hox3*, *Deformed*, *Lox5*, and *Post2*. This is fewer than the number of *Hox* genes found in the pilidiophoran *Micrura alaskensis* [16]. We were not able to isolate *Sex combs reduced*, *Antennapedia*, or *Lox4* from *P. californiensis*. These three genes are found in *M. alaskensis* [16] and are typical for other spiralians. This suggests that either these genes are not present in *P. californiensis*, are not involved in developmental processes, or are expressed at a level too low to detect at the stages we examined.

For most of the *Hox* genes examined in *P. californiensis*, expression first occurs in two lateral patches near the posterior end in the invagination-stage larvae. Later, expression appears to be restricted to the lateral nerve cords. So it appears that *Hox* genes pattern the trunk, as is the case for many other metazoans. While most *Hox* genes examined show a bilateral pattern of expression from the invagination stage onward, *PcLox5* has a non-canonical pattern of expression, in which a number of additional cells express this gene early in development. This pattern suggests that *PcLox5* may have been co-opted for some other use in early larval development, but still maintains its AP-patterning function later in development. *PcLox5* does not show non-canonical expression in the pilidiophoran *Micrura alaskensis*, so this may be a trait unique to the hoplonemerteans or to *P. californiensis*, specifically.

**Fig. 8:**
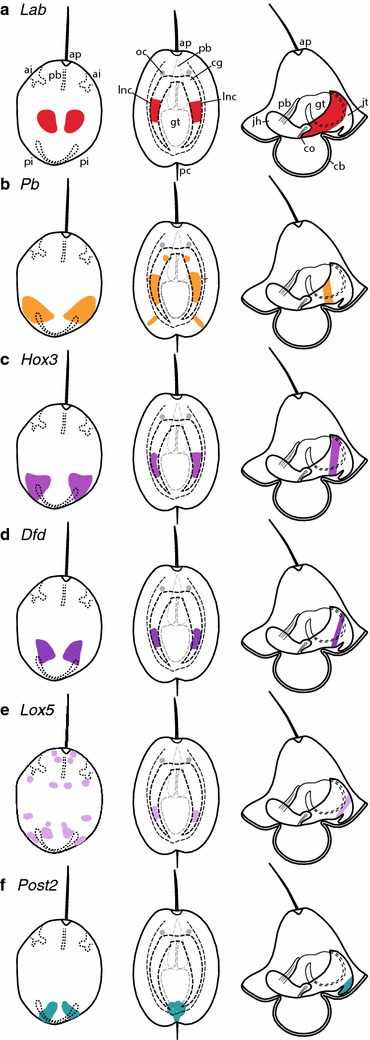
Comparison of *Hox* gene expression in hoplonemertean decidula larva and pilidium. *Diagram* illustrating expression of **a** *Lab*, **b** *Pb*, **c** *Hox3*, **d** *Dfd*, **e** *Lox5*, and **f** *Post2* in hoplonemertean decidula (invagination stage) is shown on the *left*. Hoplonemertean rudiment-stage larva in the center and pilidium on the *right*. *Ap* apical organ, *pb* proboscis, *gt* gut, *ai* anterior invagination, *pi* posterior invagination, *cb* ciliated band, *co* cerebral organ, *jh* juvenile head, *jt* juvenile trunk, *cg* cerebral ganglia, *oc* ocelli, *pc* posterior cirrus, *lnc* lateral nerve cord.

**Fig. 9:**
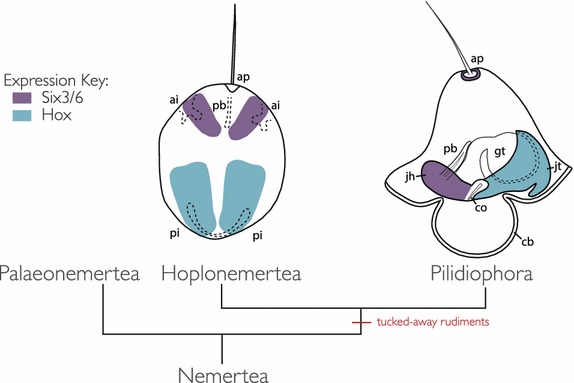
Summary diagram comparing gene expression in pilidium and hoplonemertean decidula. *Six3*/*6* (*purple*) is expressed in hoplonemertean anterior invaginations and pilidial cephalic discs and apical organ. *Hox* genes (*teal*) are expressed in hoplonemertean posterior invaginations and pilidial imaginal discs which give rise to the juvenile trunk. *ap* apical organ, *pb* proboscis, *gt* gut, *ai* anterior invagination, *pi* posterior invagination, *cb* ciliated band, *co* cerebral organ, *jh* juvneile head, *jt* juvenile trunk.

In the pilidiophoran *M. alaskensis*, the *Hox* genes pattern the imaginal discs that give rise to the juvenile trunk (paired trunk discs and the unpaired dorsal disc) (see Fig. 8) [16]. In the decidula larva of *P. californiensis*, we find that most *Hox* genes are expressed in the region adjacent to the transitory paired posterior invaginations and later on in the lateral nerve cords (see Fig. 8). Posterior invaginations have not been documented in other species of hoplonemerteans and their fate is not known in *P. californiensis* development [13], except that they appear to evaginate, at least partially, and likely contribute to the posterior definitive epidermis. Based on *Hox* gene expression patterns, we hypothesize that the decidula’s posterior invaginations may be homologous to pilidial trunk discs and give rise to the trunk tissues of the juvenile, including the epidermis and the central nervous system, specifically, the lateral nerve cords (see Fig. 9).

Paired anterior invaginations have been documented in the development of many hoplonemerteans and palaeonemerteans, but their function and fate are uncertain [see the discussion in 12, 13]. Maslakova and von Döhren [12] showed that a pair of anterior invaginations in the hoplonemertean *Paranemertes peregrina* gave rise to cerebral organs, but this was not observed in *P. californiensis* [13]. In other species of hoplonemerteans and palaeonemerteans, various authors hypothesized, but did not show definitively, that various anterior invaginations may be rudiments of various other structures, including the nervous system, cerebral organs, frontal organ, apical organ, proboscis, or the rhynchodeum [9, 15, 22–25]. We find that *Six3*/*6*, a broadly conserved gene involved in anterior neural ectoderm specification in bilaterians [26], is expressed in both the cephalic discs of *M. alaskensis* and near the anterior invaginations of *P. californiensis*. This suggests that the anterior invaginations of *P. californiensis* and, possibly, of some other hoplonemerteans and even palaeonemerteans may be homologous to pilidial cephalic discs, as was first hypothesized by Hiebert et al. [13] (see Fig. 6). Anti-serotonin antibody labeling suggests that the anterior invaginations of *P. californiensis* are associated with serotonergic neurons and may be involved in the sensory function of the larva, or the development of the adult serotonergic nervous system, or both. This supports the idea that the anterior invaginations contribute to the head region of the hoplonemertean juvenile, similar to the role of the cephalic imaginal discs in pilidiophorans, which give rise to the head of the juvenile inside of the pilidium. *Six3*/*6* expression is also observed in some cells located near the rim of the apical organ of the pilidium (Fig. 6c). No expression was observed in the apical organ of *P. californiensis* at the invagination stage, but *Six3*/*6* is expressed near the apical organ at the rudiment stage (Fig. 6b). It is parsimonious to assume that the apical plate of pilidiophorans and hoplonemerteans is homologous (since it is present in larvae of all extant nemertean taxa and was likely present in the most recent common ancestor of all nemerteans). The apical plate of the pilidium is destroyed during metamorphosis and does not participate in the formation of the adult. Similarly, the apical plate in hoplonemerteans is gradually remodeled during transition to the juvenile life (adults have no apical plate). In *P. californiensis*, the expression exists in two broad lateral domains at the invagination stage in regions that appear to persist through to juvenile stages, which is more similar to the cephalic-disc expression in the pilidium than the apical organ expression. Thus, we are fairly certain that the appropriate comparisons should be: first, between the expression in the apical plates of both species; and second, between the expression in the anterior invaginations in the hoplonemertean development and the cephalic discs in the pilidium.

Developmental homology between pilidial imaginal discs and invaginations of the decidula larva is also supported by the patterns of cell division. Anti-phospho-histone antibody labeling shows that the two pairs of invaginations in the hoplonemertean larva contain many dividing cells. Thus, hoplonemertean larval invaginations are sites of cell proliferation, much like the axils and the imaginal discs in the pilidium [27]. One might speculate that it may be functionally advantageous to tuck away the growth zones into some sort of invaginated rudiment(s) of a swimming larva. The decidula’s epidermis comprises multiciliated cells, which cannot divide [see discussion in 27]. If proliferative zones which consist of non-ciliated or monociliated cells were left on the surface, they would disrupt the pattern of ciliation and thus might affect larval swimming.

The finding that pilidial imaginal discs have a likely homolog in the hoplonemerteans has important implications for the origins of the pilidium. These results suggest that invaginated rudiments of some sort were likely present in the hoplonemertean–pilidiophoran ancestor. We suggest that the decidula’s invaginations may be more representative of the ancestral condition than the pilidial imaginal discs, because the presence of the imaginal discs in the ancestral larva would require a severe reduction of pilidial features within the Hoplonemertea. Supporting this case is the fact that the typical planktotrophic pilidium appears to have been lost several times in pilidiophoran evolution, being replaced with highly reduced lecithotrophic forms [11, and references therein]. All studied secondarily reduced pilidia retain imaginal discs and catastrophic metamorphosis [28–30]. If the hoplonemertean–pilidiophoran ancestor was more pilidium-like, we would expect that more prominent imaginal discs and metamorphosis would be present in the extant hoplonemerteans.

## Conclusions

*Hox*, *Cdx*, and *Six3*/*6* gene expression as well as cell proliferation patterns support the homology between pilidial imaginal discs and hoplonemertean larval invaginations (Fig. 7). This suggests that the common ancestor of pilidiophorans and hoplonemerteans likely developed tucked-away juvenile rudiments of some sort. Invaginated rudiments in the hoplonemertean–pilidiophoran ancestor may represent the precursors to imaginal discs in the pilidium.

## Additional files

Additional file 1: Tables summarizing sequence data. Additional file 2: Bayesian phylogenetic analysis including *P. californiensis* *Hox* genes. Bf; *Branchiostoma floridae* (Cephalochordata), Tc; *Tribolium castaneum* (Arthropoda), Dm; *Drosophila melanogaster* (Arthropoda), Ct; *Capitella teleta* (Annelida), Bt; *Bugula turrita* (Bryozoa), Ls; *Ramphogordius *(*Lineus) sanguineus* (Nemertea), La; *Lingula anatina* (Brachiopoda), Es; *Euprymna scolopes* (Mollusca), Ma; *Micrura alaskensis* (Nemertea), Pc; *P. californiensis* (Nemertea). Numbers above clades indicate Bayesian posterior probabilities higher than 50%. Colors denote paralogy groups (PGs). Arrowheads indicate *Pantinonemertes californiensis* *Hox* genes isolated by us. Scale bar, 0.7 nucleotide substitutions/site. Additional file 3: Bayesian phylogenetic analysis including *P. californiensis* and *M. alaskensis* *Six* family genes. Mm; *Mus musculus* (Chrodata), Ct; *Capitella teleta* (Annelida), Dm; *Drosophila melanogaster* (Arthropoda), Sk; *Saccoglossus kowalevskii* (Hemichordata), Sp; *Strongylocentrotus purpuratus* (Echinodermata), Nv; *Nematostella vectensis* (Annelida), Tt; *Terebratalia transversa* (Brachiopoda), Pd; *Platynereis dumerilii* (Annelida), Pc; *Pantinonemertes californiensis* (Nemertea), Ma; *Micrura alaskensis* (Nemertea), Lg; *Lottia gigantea* (Mollusca). Numbers above clades indicate Bayesian posterior probabilities higher than 50%. Arrowheads indicate nemertean *Six3/6* genes isolated by us. Scale bar, 0.2 nucleotide substitutions/site.
