# Supplementary material for: Expression of Hox, Cdx, and Six3/6 genes in the hoplonemertean Pantinonemertes californiensis offers insight into the evolution of maximally indirect development in the phylum Nemertea
Source: EvoDevo. 2015 Aug 4;6:26. doi: 10.1186/s13227-015-0021-7 (PMC4524027; doi:10.1186/s13227-015-0021-7)
Supplement: Additional file 1: — Tables summarizing sequence data. [file 13227_2015_21_MOESM1_ESM.docx]

## Supplementary Table 1 - Sequences used for Hox alignment and phylogenetic analysis

| Species | Gene names | Accession # |
| --- | --- | --- |
| Branchiostoma floridae | Bf_hox1 Bf_hox2  Bf_hox3  Bf_hox4  Bf_hox5 Bf_hox6 Bf_hox7 Bf_hox8 Bf_hox9 Bf_hox10 Bf_hox11 Bf_hox12 Bf_hox13 Bf_hox14  Bf_cdx | BAA78620 BAA78621  X68045  BAA78622 CAA84517 CAA84518 CAA84519 CAA84520 CAA84521 CAA84522 AAF81909 AAF81903 AAF81904 AAF81905  AAC39017 |
| Bugula turrita | Bt_pb Bt_hox3 Bt_dfda Bt_dfdb  Bt_lox5  Bt_post2 | AAS77225  AAS77226  AAS77227  AAS77228  AAS77229  AAS77230 |
| Capitella teleta | Ct_lab  Ct_pb  Ct_hox3  Ct_dfd  Ct_scr  Ct_lox5  Ct_antp  Ct_lox4  Ct_lox2  Ct_post1  Ct_post2  Ct_eve  Ct_cdx | ABY67952  ABY67953  ABY67954  ABY67955  ABY67956  ABY67957  ABY67962  ABY67958  ABY67959  ABY67961  ABY67960  ABG82164  AAZ95508 |
| Drosophila melanogaster | Dm_lab Dm_pb  Dm_zen  Dm_dfd Dm_scr Dm_ftz Dm_antp Dm_ubx Dm_abdA Dm_abdB  Dm_eve  Dm_cdx | CAB57787  CAA45271  P09089  P07548  NP_524248  NP_477498  CAA27417  CAA29194  P29555  CAB57859  AAF58865  AAA28409 |
| Euprymna scolopes | Es_lab Es_hox3 Es_scr Es_lox5 Es_antp Es_lox4 Es_post1 Es_post2  Es_cdx | AY330184  AY330185  AY330186  AY330187  AY330188  AY330189  AY330190  AY330191  AF127341 |
| Lingula anatina | La_lab La_hox3 La_scr La_lox5 La_antp La_lox2 La_lox4 La_post2 La_post1 | AAD45587  AAD45588  AAD45589  AAD45591  AAD45590  AAD45592  AAD45593  AAD45595  AAD45594 |
| Lineus sanguineus | Ls_hox1 Ls_hox3  Ls_hox4  Ls_hox6  Ls_hox7  Ls_hox9 | CAA76295 CAA76296  P81192  CAA76297 CAA76298 CAA76299 |
| Tribolium castaneum | Tc_lab  Tc_pb  Tc_zen  Tc_dfd  Tc_scr  Tc_ftz  Tc_antp  Tc_ubx Tc_abdA Tc_abdB  Tc_eve  Tc_cdx | NP_001107762  EEZ99256  NP_001036813  NP_001034510  AAK16422 NP_001034539  EEZ99250  NP_001034497 NP_001034518 NP_001034519  NP_001034538  NP_001034498 |
| Micrura alaskensis | Ma_lab  Ma_pb  Ma_hox3  Ma_dfd  Ma_scr  Ma_antp  Ma_lox5  Ma_lox4  Ma_post2  Ma_cdx | KP762174 KP762176 KP762173 KP762180 KP762177 KP762171 KP762179 KP762175 KP762178 KP762170 |

## Supplementary Table 2 - Sequences used for Six alignment and phylogenetic analysis

| Species | Gene names | Accession # |
| --- | --- | --- |
| Mus musculus | Six1  Six2  Six3  Six3/6  Six3/6  Six4  Six5  Six6 | NP_033215  NP_035510  NP_035511  ELU17224  ELU17226  NP_035512  NP_035513  NP_035514 |
| Capitella teleta | Six1/2  Six3/6  Six4/5 | ELU17231  ELU17225  ELT94068 |
| Drosophila melanogaster | optix  Six4  sine-oculis | NP_524695.2  NP_649256  NP_476733 |
| Saccoglossus kowalevskii | Six2  Six3  Six4 | XP_002735213  NP_001158378  XP_002735606 |
| Strongylocentrotus  purpuratus | Six1  Six3/6  Six4 | XP_001181583  XP_781696  XP_001181543 |
| Nematostella vectensis | Six1/2  Six3/6 | XP_001626434  XP_001625159 |
| Terebratalia transversa | Six3/6 | AEZ03831 |
| Platynereis dumerilii | Six2  Six3 | CAC86663  CAR66435 |
| Pantinonemertes californiensis | Six3/6 | KP762169 |
| Micrura alaskensis | Six3/6 | KP762172 |
| Lottia gigantea | Six1/2  Six3/6  Six4/5 | ESO86170  ESO86168  ESO96422 |

## Supplementary Table 3 - *Hox* genes isolated from *P. californiensis*

| **Paralogy group** | **Gene name** | **Probe length** | **Predicted ORF** | **Genbank accession #** |
| --- | --- | --- | --- | --- |
| PG1 | PcLab | 1685bp | 882bp | KP762168 |
| PG2 | PcPb | 1736bp | missing 5’ end | KP762165 |
| PG3 | PcHox3 | 1171bp | 951bp | KP762164 |
| PG4 | PcDfd | 1193bp | 708bp | KP762163 |
| PG6 | PcLox5 | 1600bp | 735bp | KP762162 |
| PG9-15 | PcPost2 | 1198bp | 1029bp | KP762166 |
|  | Cdx | 985bp | 427bp | KP762167 |
